# Bistability and Bacterial Infections

**DOI:** 10.1371/journal.pone.0010010

**Published:** 2010-05-05

**Authors:** Roy Malka, Eliezer Shochat, Vered Rom-Kedar

**Affiliations:** 1 Department of Computer Science and Applied Mathematics, The Weizmann Institute of Science, Rehovot, Israel; 2 Pharma Research and Early Development, Hoffmann-La Roche, Basel, Switzerland; 3 The Estrin Family Chair of Computer Science and Applied Mathematics, Department of Computer Science and Applied Mathematics, The Weizmann Institute of Science, Rehovot, Israel; Vrije Universiteit, Netherlands

## Abstract

Bacterial infections occur when the natural host defenses are overwhelmed by invading bacteria. The main component of the host defense is impaired when neutrophil count or function is too low, putting the host at great risk of developing an acute infection. In people with intact immune systems, neutrophil count increases during bacterial infection. However, there are two important clinical cases in which they remain constant: a) in patients with neutropenic-associated conditions, such as those undergoing chemotherapy at the nadir (the minimum clinically observable neutrophil level); b) in ex vivo examination of the patient's neutrophil bactericidal activity. Here we study bacterial population dynamics under fixed neutrophil levels by mathematical modelling. We show that under reasonable biological assumptions, there are only two possible scenarios: 1) Bacterial behavior is monostable: it always converges to a stable equilibrium of bacterial concentration which only depends, in a gradual manner, on the neutrophil level (and not on the initial bacterial level). We call such a behavior type I dynamics. 2) The bacterial dynamics is bistable for some range of neutrophil levels. We call such a behavior type II dynamics. In the bistable case (type II), one equilibrium corresponds to a healthy state whereas the other corresponds to a fulminant bacterial infection. We demonstrate that published data of in vitro *Staphylococcus epidermidis* bactericidal experiments are inconsistent with both the type I dynamics and the commonly used linear model and are consistent with type II dynamics. We argue that type II dynamics is a plausible mechanism for the development of a fulminant infection.

## Introduction

The human body is constantly exposed to bacterial influx from the environment via the skin, the respiratory tract and the digestive organs. At the acute stage of bacterial infection, the neutrophils (phagocytic white blood cells), which make up about 70% of the white-cell blood count in adults, are the main cells that fight the bacteria. The environmental conditions, neutrophil level and efficiency of the human barrier tissues play a crucial role in the susceptibility of the human body to infections [1]. There are several known medically significant conditions of neutrophils with reduced number or function that are associated with an increased risk of infection: patients with severe neutropenia (neutrophil count less than 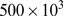 neutrophils/mL in the blood, which is three to ten times less than the normal values) [2], [3]; people suffering from impaired microbicidal machinery (such as chronic granulomatous disease-CGD) [4], [5]; individuals with neutrophil-adhesion deficiency (which prevents the neutrophils from leaving the blood vessels and reaching the site of infection) [6]; individuals with insufficient vasculature to deliver neutrophils to the site of infection (e.g. deep burns) [7]. We refer to these medical conditions as *neutropenia-associated conditions*. In the medical literature neutropenia-associated conditions refer to reduced number of neutrophils and is separated from neutrophils malfunction, here we bind them together for simplicity of presentation. These full-body conditions seem to establish the notion that there exists a critical neutrophil concentration below which the risk of infection dramatically increases. These observations motivated several groups to perform in-vitro experiments, with the notion that characterizing the bacterium-phagocyte dynamics would help decipher the in-vivo behavior of the innate immune system.

Two views regarding the possible in vitro dynamic behavior of the bacteria emerged from these experiments. Clawson and Repine, Leijh et al. and Hammer et al. [8]–[10] proposed that bacterial killing by neutrophils is ratio-dependent. In their experiments, the neutrophil concentration was fixed (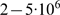 neutrophils/mL) and neutrophil-bacteria ratios of 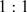 to 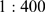 were achieved by varying the initial bacterial concentration. On the other hand, Li et al. [11], [12] proposed that there is no ratio dependency, and that there exists a unique critical neutrophil value: below this value, the neutrophils cannot control the bacterial growth at all, regardless of bacterial concentration, and above this value, the neutrophils can control any bacterial concentration. They further proposed that the value of this critical neutrophil concentration can be estimated from a simple linear mathematical model that is fitted to the experimental data. This estimated critical value agrees with the commonly accepted in-vivo critical value for severe neutropenia. Here we further develop this notion of critical neutrophil concentration and show, by mathematical considerations, that near this critical value, non-linear effects cannot be ignored even at small bacteria concentrations.

Mathematical modelling of the immune system has a long and rich history, mainly in the context of the adaptive immune response. There, ordinary differential equations (ODEs) describing spatially homogeneous population dynamics are commonly used (see [13]–[15] and references therein). Indeed, the model proposed here, as well as Li et al. [11], [12] linear model, belong to the class of predator-prey models in which the predator population is fixed. The linear version is just the prey equation of the Lotka-Volterra system (see e.g., [16, p.79]). Non-linear effects and the natural emergence of bistability have been widely studied in this context, see e.g., [17], [ 18, p.74]. Moreover, it has been observed that bistability can be experimentally verified by taking advantage of the hysteresis effect [19]–[21].

Models for studying the innate immune response in general and the phagocyte-bacterium interactions in particular, are scarce. Most studies have concentrated on various in vivo medical conditions, hence these are inherently of higher dimension, and always include an equation that describes the bacteria dynamics coupled to the phagocyte concentrations. Most commonly, the normal to hyper-response of the phagocytes to the invasion of bacteria is studied. In Kumar et al., Chow et al. and Reynolds et al. [22]–[25], the focus is on the relation between the inflammatory response, anti-inflammation mediators and sepsis. In Herald [26], the macrophage dynamics is related to the development of chronic inflammation after eradication of a pathogen. In Pugliese and Gandolfi [27], the in-vivo pathogens and specific and non-specific immunity dynamics are shown to be potentially quite rich: bistable regions and oscillatory regimes appear as the model parameters are varied. Imran and Smith [28] consider the influence of bacterial nutrients on the innate immune response to bacterial infection and identify locally stable disease-free region, which is used to design a successful antibiotics treatment. Finally, models concentrating on in vivo blood-tissue dynamics showed good fit to experimental data of Escherichia coli concentration in milk produced from infected cows [29].

Early modelling efforts of bacterium-phagocyte dynamics in vitro [30], [31] focused on probabilistic modelling of the number of digested particles per neutrophil. These issues were further explored experimentally, and led to the proposal of a three-compartment linear ODE model in which the dynamics of viable, phagocytosed and perforated bacteria are presented [32]. The works of Li et al. [11], [12] were the first to relate the implication of such experiments and models to the observed in-vivo critical value of neutrophils.

Notably, in mathematics, as in biology, characterization of a simplified system (such as an in-vitro experiment) serves as a building block for more complex systems. The mathematical building block of Li et al. [11] was utilized in several models of in-vivo dynamics, e.g., [29], [33], whereas others modified this part of the model and included non-linear and saturation effects (see e.g. [27]). Thus, our work may be viewed as a careful construction of an essential building block of more complex systems (see discussion).

Below we briefly describe a class of mathematical models for bacterial growth when neutrophils are present. We show that non-linear models that respect elementary robust biological observations can be divided into exactly two types of behavior that we call type I and type II. In the Results section, we show that the data in Li et al. [12] provide experimental evidence that **falsifies** the adequacy of the type I and linear behaviors, yet corroborates the adequacy of type II behavior, (We purposely avoid the word “validate”, taking the Popperian [34] view that scientific theories may only be falsified by experiments). In particular, we show that the dynamics is not ratio-dependent, and the critical neutrophil value does depend on the initial bacterial concentration. We conclude with some observations regarding the clinical implications of these findings.

## Methods

### Modelling the Bacterium-Phagocyte Dynamics

To model the bacterial dynamics in a suspension, we write an ordinary differential equation for the rate of change of the bacterial concentration 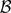 [bacteria/mL], and describe how this rate depends on the neutrophil concentration 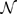 [neutrophils/mL] and on the external bacterial influx, Infx [bacteria/mL min]. Such models are adequate for describing well-mixed large population dynamics. For the clinically relevant values (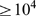 cells/mL) stochastic effects may be neglected (see e.g. [35]). Our aim here is to construct such a model from first principles, so that the qualitative dynamic consequences will depend only on the biological assumptions that enter the model and will not depend on the detailed functional form of the equations.

To this aim, the rate of change of the bacterial concentration is divided into bacterial birth terms (natural growth and influx) and death terms (bacterial natural death and killing by neutrophils):
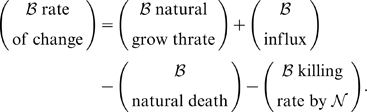



Below, we list our assumptions on the form of each of the above terms. In particular, these considerations imply that each of the non-constant terms is a monotonic function of 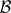 (and the killing term is also monotone in 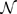). Moreover, we always assume that the growth/death rates (namely, terms on the right hand side of the equation divided by 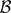) are saturable as these are controlled by biological processes that always have a finite maximal rate.

 **A1.** The natural bacterial dynamics (with no neutrophils) has a limited growth curve.
  Namely, by this assumption, the sum of the first two terms is larger than the third term for 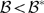 and the opposite statement holds for 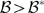, where 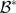 denotes the maximal capacity state (also known as the carrying capacity in the logistic model). Indeed, it is observed that in well-mixed suspensions, small concentrations of bacteria grow till they reach high concentrations and then the density asymptotes to a natural stable equilibrium, the maximal capacity state. Various mathematical models that fit experimental growth curves (e.g. logistic, Gomperz, and saturated growth models such as eq. (1)) satisfy this assumption (see [36]).
 **A2.** Sufficiently large neutrophil concentrations defeat small bacterial concentrations.
  This assumption presents the minimal requirement for the ability of neutrophils to control bacteria as observed in the lab and in the clinic (see e.g. [10]). This assumption holds for both gram-positive and gram-negative bacterial strains provided that in the gram-positive case, there is an ample supply of opsonins [9].
 **A3.** The kill rate increases and is saturable with both 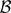 and 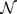.
  The monotonicity assumption, namely that the more neutrophils or bacteria we have, the larger the killing, is plausible for any killing-term form. The saturability effects in both 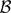 and 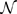 (as in the Michaelis-Menten type terms that appear in predator-prey models with predator interference) [37], [38] also have a clear biological motivation: each neutrophil has a limited killing capacity so the killing term is saturable in 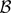 [9]. At high concentrations of 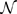 (for a fixed 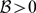), the availability of bacteria per neutrophil decreases and thus the killing term is saturable in 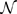. At limited 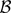 values, this assumption is valid for all bacterium-phagocyte dynamics, see the Discussion section.
 **A4.** The rate of change of 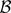 does not depend explicitly on time or space, or on any other dynamic variable (so in particular, the neutrophil level 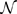 and the 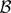 influx Infx are non-negative constants).
  This assumption is reasonable for standard in-vitro experiments and lab tests in well mixed suspensions for a limited time scale of about 90 min (so that the neutrophil function and number are not affected, nor is any other aspect of the medium that influences the natural bacterial growth, see e.g. [8], [11], [39]). The possible applicability of A4 to specific in-vivo situations is a non-trivial issue, see the Discussion section.

Most of the results described in this paper apply to all models that satisfy the robust biological assumptions A1–A4. However, for concreteness and simplicity of presentation, we hereafter consider the following explicit model:
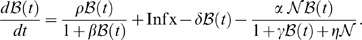
(1)


This model satisfies assumptions A1–A4 for sufficiently small bacterial influx (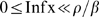) provided the six parameters 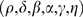 are all positive and satisfy 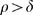 (the maximal capacity here is 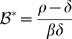) and 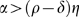. The first three parameters govern the natural dynamics of the bacteria: 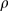 and 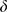 control the natural linear growth/death rates of the bacteria and 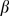 controls the natural saturation of the bacterial growth rate at high concentrations (this term reduces to the commonly used logistic term when 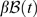 is sufficiently small). The other three parameters control the killing of the bacteria by the neutrophils: 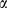 is the neutrophils' bacterial killing rate at low concentrations, and 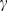 and 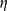 control the saturation in the killing rate as the concentrations of 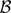 and 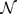 are increased (see A3). All of these parameters depend on both the bacterial strain and the environmental setting: for example, in an experimental set-up, the serum content and the form of the toxic clearance affect them. In fact, these parameters represent global effective responses to a large complex network of molecular and cellular processes that are associated with the bacteria natural growth and with the capture and killing of the bacteria by neutrophils. A calibration of these parameters with extensive experimental data and with the corresponding molecular markers may be important for identifying the main effect of these cellular processes on the bacteria-neutrophil population dynamics in health and in disease.

This model is non-linear–it takes into account the saturation effects that appear at high concentrations. If these effects are neglected, and the influx is set to zero (so 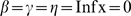), we arrive at the linear model: 
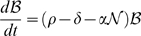
, (see e.g. [11]). This linear model has, for all 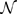 only a single equilibrium at the origin. This fixed point is unstable for small 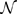 and stable when 
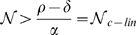
 (as in [11]). Thus, the transient bacterial dynamics is always independent of the initial bacterial concentration: if 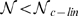 (respectively 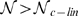), the bacterial population grows exponentially without bound (respectively shrinks exponentially to zero). Moreover, the exponential rate of transient growth/decay of the bacterial population depends only on the neutrophil concentration: it is independent of the initial positive concentration of the bacteria.

Notice that this linear model fails to satisfy assumptions A1 and A3. One may postulate that since saturation occurs only at high concentrations (near 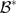), the linear model will adequately describe the dynamics at smaller concentrations. Next, we show that near 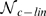 this postulate is false: the dynamics in the non-linear and linear models are substantially different, even when 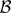 is small.

## Results

### Two Possibilities: Robust Dynamics vs. Bistable Dynamics

Mathematical analysis of Eq. (1) shows that depending on the parameters, one of exactly two types of behaviors, called hereafter type I and II, can occur (a similar statement can be made for all models satisfying assumptions A1–A4.).

**Figure 1:**
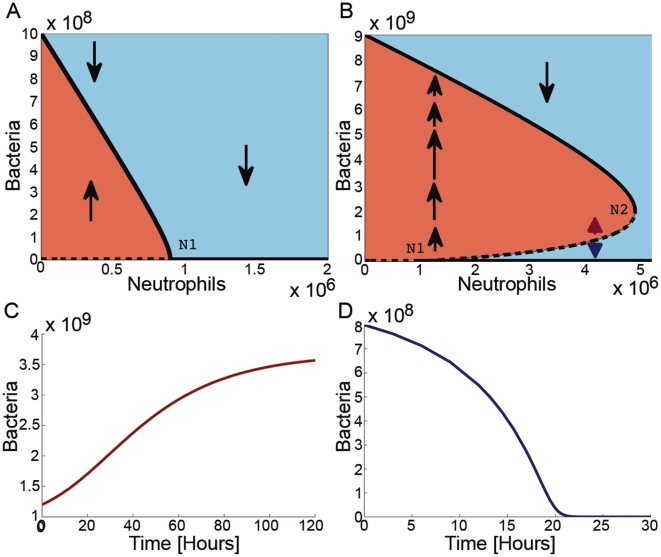
The bifurcation diagram: the bacterial equilibrium points (EPs) as a function of neutrophil concentration. Solid line indicates a branch of stable EPs, and dashed line indicates a branch of unstable EPs. (**a**) Type I dynamic has a unique stable branch of EPs for all 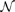 values. The black arrows demonstrate that for any positive initial value of the bacteria, for any 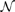, 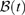 converges to the corresponding unique stable EP (the intersection of the solid black curve with a vertical line). Bifurcation diagram is drawn for Eq. (1) with 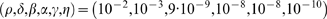 (**b**) Type II dynamic has a region of bistability: when 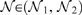, the final state of 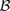 depends on whether the initial bacterial concentration is above or below the critical bacterial curve of unstable EPs (dashed line). The bifurcation diagram is drawn for Eq. (1) with 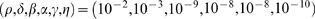. (**c–d**) Time plots of the two initial bacterial concentrations notated by red up and blue down arrows in (b) for a fixed 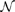 value (notice the different time scales).

**Figure 2:**
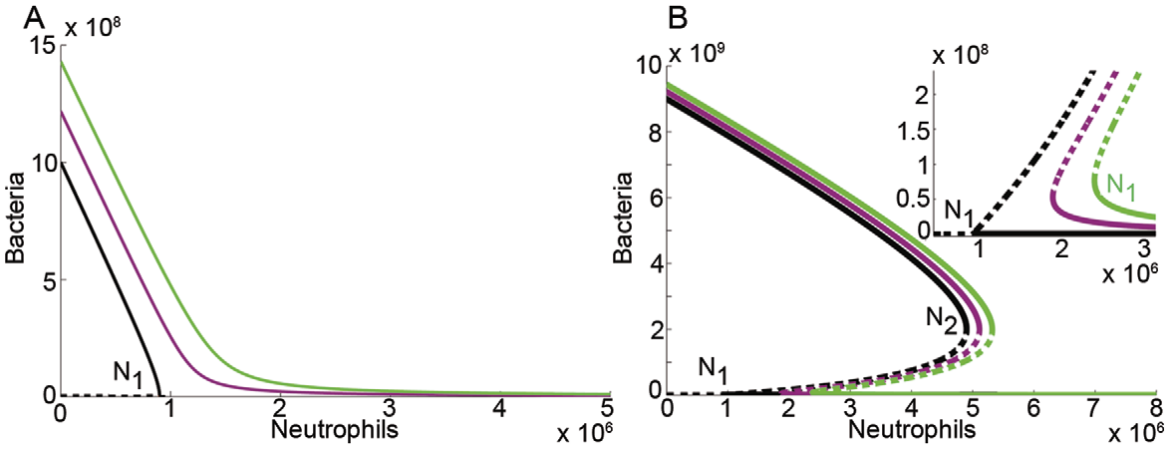
Bifurcation diagrams with zero and small positive bacterial influx. Bifurcation curves for Infx  = 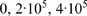 are shown in black, magenta and green, respectively. When Infx 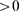, the bifurcation curves are shifted to the right. (**a**) Type I: the zero Infx transcritical bifurcation point at 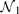 disappears when 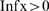. (**b**) Type II: the zero Infx transcritical bifurcation becomes a saddle-node bifurcation that appears at a distance 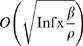 from the transcritical bifurcation point (see Bacterial Influx). (**Insert**) A close-up around the transcritical bifurcation of (b). This diagram shows that under perturbation (Infx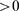), the transcritical bifurcation becomes a saddle-node bifurcation.

In the type I parameter regime, the model has no *critical* dependence on neutrophil concentration 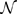. That is, for all levels of neutrophils there is a single stable equilibrium point (EP) which depends gradually on 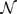: for low values it corresponds to the high concentration point associated with the maximal capacity branch–the branch of stable equilibria that emanates from the point 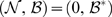, where 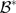 is the maximal capacity state of the natural bacterial dynamics. As the neutrophil level increases, this EP gradually lowers till, for sufficiently high neutrophil level (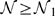), it reaches the origin (see Fig. 1a and Models section; for simplicity of presentation, we consider here the zero influx and show in the Bacterial Influx section that the results are only slightly modified for small bacterial influx, see also Fig. 2).

In the type II parameter regime, the model exhibits bistability for neutrophil concentrations between levels 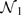 and 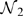 (see Fig. 1b). For neutrophil levels below 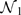, small bacterial concentrations always grow to the maximal capacity branch. For neutrophil levels in the range 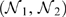, the neutrophils can overcome *some* portion of the bacterial population but not all of it (see Fig. 1b). Therefore, in this range, there exists a critical bacterial concentration, 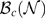, above which bacterial growth dominates and below which the neutrophils take control (see dashed curve in Figs. 1b, 2b). Moreover, in this range the critical curve 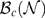 is a non-linear increasing function of the neutrophil count 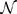. Summarizing, 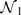 distinguishes between neutrophil levels that cannot control *any* non-trivial initial population of the bacteria and levels that can control a *limited size* of the initial bacterial population. A further increase in neutrophil levels beyond 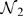 again leads to robust dynamics by which the neutrophils can control any size of bacterial population (see Fig. 1b). This regime, of complete robustness, appears in the ideal in-vitro setting, where the bacterial natural growth is limited and the neutrophils concentration and function is kept constant. Then, very high initial concentrations of bacteria naturally decrease to the maximal capacity concentration, and so, if there are enough neutrophils to overcome the maximal capacity, they indeed defeat any size of bacterial infection. This part of the dynamics is expected to be usually irrelevant to the in-vivo dynamics due, for example, to the neutrophil toxicity (see model limitation section).

To succinctly present the differences between the type I and type II behaviors, we plot representative bifurcation diagrams (Fig. 1)– diagrams that show the equilibrium points dependence on 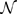. It is important to note that these bifurcation diagrams also explain how the *transient* behavior depends on the initial concentrations in the different regimes. Indeed, the bifurcation diagrams help us divide the 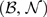 plane into regimes of qualitatively different transient behaviors: regimes in which the bacterial concentration grows vs. regimes in which it decays. Notably, such predictions regarding the different transient regimes may be tested by the standard 90-min bactericidal-phagocyte experiments (see the Published Data section).

**Figure 3:**
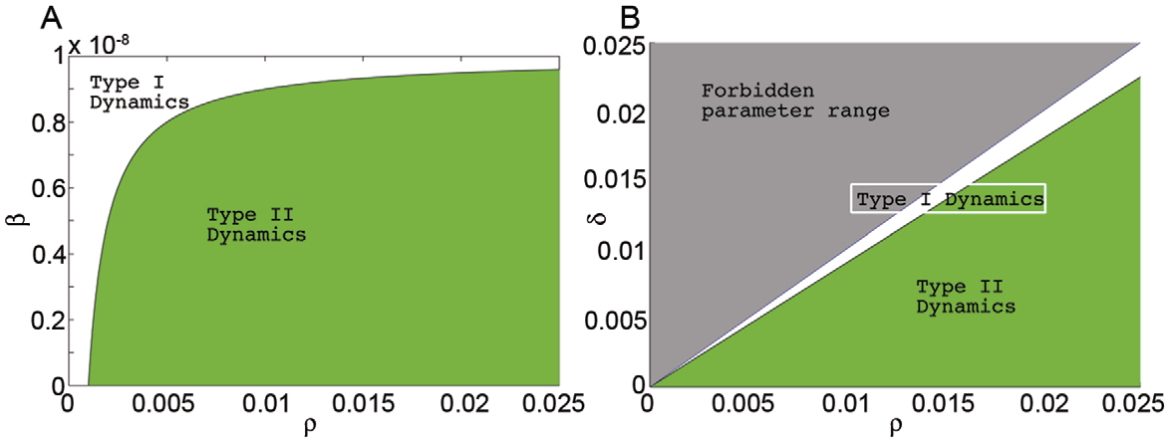
The natural bacterial parameter space division into type I and type II dynamics. The behavior type is found from the derived analytical conditions (see Methods) for fixed killing-term parameters as in Fig. 1 (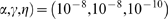. Notably, most of the parameters give rise to type II behavior. (**a**) The 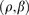 space is shown for 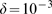. (**b**) The 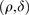 space is shown for 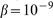. The grey region 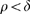 is forbidden, as it corresponds to a reduction in the bacterial population even with no neutrophils, violating A1.

Finally, note that the parameter regimes that determine the resulting type of behavior depend on the bacterial strain, on neutrophil function and on the environmental factors (see Models for the exact expression). For example, by increasing the bacteria's nutrient supply, the bacterial dynamics will change from type I behavior under very poor conditions, to type II dynamics for a sufficiently rich environment (see Fig. 3 for the separation of the two behaviors in parameter space).

Notably, only the type II dynamics admits the hysteresis effect; starting with a neutrophil level 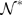 in the interval 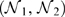 and a tiny bacterial population that is under control (representing a healthy person), a slow decrease in 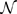 followed by a slow increase in neutrophils back to the original value 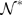 (due, e.g., to a chemotherapy treatment), may result in subsequent bacterial growth that may lead to a fulminant infection. The same cycle of 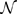 with the type I dynamics or the linear model will always eventually result in full recovery. We thus propose that while there are strains of bacteria exhibiting both types of dynamics in the body, those that exhibit type II dynamics are the main contributors to the onset of acute infections (see Discussion). We next show that type II dynamics indeed appear in nature.

### Experimental Evidence for Bistability

Here we analyze the results published by Li et al. [12] of bactericidal experiments, and show that the published data correspond to type II bacterial dynamics rather than to type I or linear dynamics.

Our analysis of the type II model predicts the existence of a critical bacterial curve, 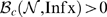 (dashed branch in Fig. 1b) at which the rates of growth and killing exactly balance, yet this balance is unstable. This sensitive dependence of the dynamics on initial conditions presents the fundamental difference between the type II behavior and the linear and type I behaviors. This dependency appears only in the type II parameter regime and only in the interval 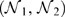 where the critical bacterial curve separates between initial conditions of bacterial concentrations that decay towards the stable healthy state (the lower equilibrium curve) and those that grow towards the infectious state (the upper equilibrium curve).

We show next that the data of the bactericidal experiments [12] support the existence of such a critical bacterial curve.

#### Published Data

**Figure 4:**
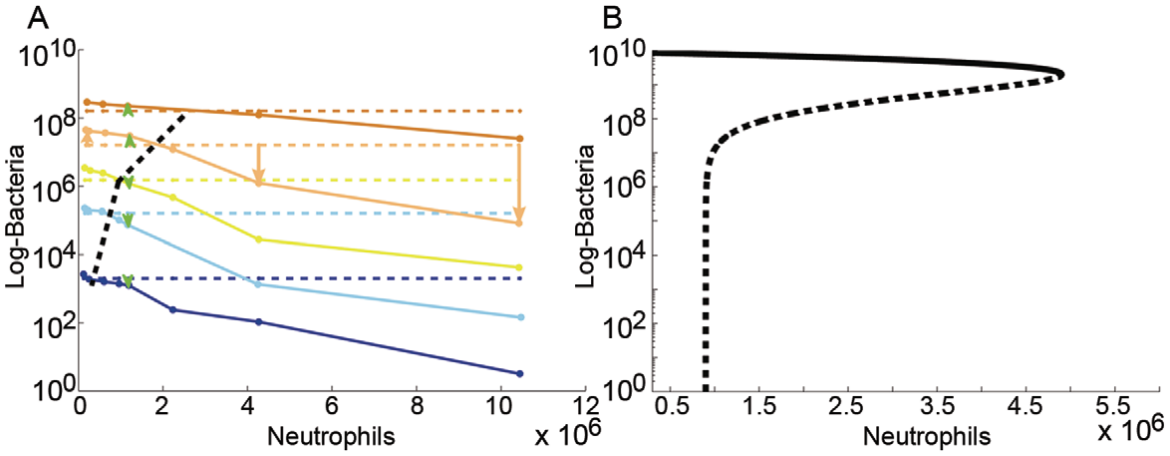
Experimental support for the model prediction of bistability. (**a**) A phase-space presentation of the data from Li et. al. [12]. In this experiment, neutrophils and *S. epidermidis* bacteria were added into a gel and the bacteria were recovered after 90 min. The tail of each of the arrows indicates the bacterial concentration at 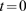 and the head indicates the concentration at 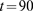 min. Dashed colored lines: the initial bacterial concentrations. Solid colored lines: the corresponding final bacterial concentrations (connecting the corresponding data points). The black bold-dashed line is the estimated critical curve between neutrophil killing and bacterial growth rate. (**b**) The type II model (Fig. 1b) bifurcation diagram in logarithmic scale.

In the experiments by Li et al. [11], [12], 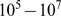 neutrophils/mL, and 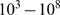 CFU/mL of *S. epidermidis* bacteria were added into a suspension or a fibrin gel, simulating human blood and tissue, respectively. The bacterial level was then recovered from the suspension/gel after 90 min. Fig. 4a presents the data from Li et al. (Fig. 3b in [12]) in a different way, namely in the 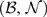 plane. Each *colored horizontal* dotted line connects the experiments with identical initial bacterial levels. These horizontal lines are mapped, after 90 min, to the solid curves of the same color, now connecting the final data points of the experiments that started with identical initial bacterial concentration; for clarification, some are emphasized by arrows: each arrow indicates the bacteria at 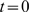 (tail) and at 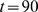 min (head) for the relevant neutrophil concentration level. When looking at a fixed 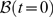 for increasing 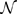 values, the arrows (see e.g. the dotted orange horizontal line and the orange, green, and two orange arrows in Fig. 4a) decrease in size, till they change direction, and then they increase again in the opposite direction (i.e., 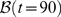 is decreasing with the increase in 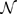). From these two properties (size and direction) we conclude that there is a curve of “zero-sized arrows”, i.e., points of balance between natural bacterial growth and their killing by neutrophils–these points belong to the approximated black bold dashed curve in Fig. 4a. Looking at a fixed 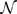 for increasing 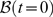 values (vertical direction in Fig. 4a) shows that for low initial bacteria below the dashed-black curve, the bacterial population is decreasing (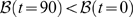, arrow down) and for high initial bacteria, above this line, the population is increasing (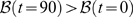, arrow up). This experimentally derived curve (dashed line in Fig. 4b) corresponds to the predicted critical bacterial curve 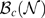 (dashed line in Fig. 1b). Thus, the existence of a positive unstable equilibrium curve 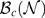 for a range of 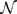 values is now established–for the experimental setting in [12] we now know that 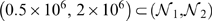. Such behavior is consistent with type II dynamics and is inconsistent with either the linear or type I dynamics. We conclude that the results presented in Fig. 4a refute the validity of linear and type I dynamics: in these two cases, one may not change the transient behavior of the bacteria from decreasing to increasing by elevating the initial bacterial concentration as occurs at 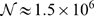 (see green arrows in Fig 3a). These experimental results corroborate the theoretical prediction that the in-vitro dynamics exhibits a type II behavior.

## Discussion

A model of bacterial dynamics interacting with a constant level of neutrophils and a constant influx term was constructed. Two distinct dynamic behaviors, identified as type I and type II, emerged. In the type II regime, the bacterial dynamics exhibits bistability for a range of neutrophil levels. We showed that published experiments do exhibit such bistability, namely that type II dynamics do appear in in vitro experiments of bacterial growth under phagocyte attack.

### Plausible Clinical Implications

Our modelling effort has two objectives: first, to describe the dynamics of bacteria interacting with constant levels of neutrophils in vitro and second, to gain some insights on the role of neutrophil levels and barrier integrity at the onset of bacterial infection in humans.

We first stress that in vitro studies are clinically important: these are widely used to estimate neutrophils functionality in patients with repeated infections (e.g., to diagnose neutropenia-associated conditions such as the malfunctioning of the neutrophils in CGD patients [4]). Our findings suggest an additional perspective on some of the bactericidal tests that are performed in clinical immunology labs [4]. In these tests, the neutrophil killing functionality for a given ratio between the bacteria and the neutrophils (e.g. 1∶2 ratio of 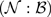) is recorded, where neutrophil function in patients and healthy controls are compared, and multiple wells are used for quality assurance. The common wisdom among clinicians is that these tests are unreliable when the actual concentrations are too low. We can now explain this: at low concentrations, the tests are probably performed near the critical bacterial curve, and near this curve very small experimental inaccuracies can push one well to be above the critical curve and the other to be below it–leading to substantial deviation in the results that are thus interpreted as unreliable data. Notably, this experimental experience again supports the emergence of type II dynamics in such clinical evaluations: type I behavior does not support sharp transitions at any given 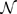, and thus predicts small error bars between different experiments, contrary to the clinical observations at small concentrations. Thus, we conclude that one needs to fit a non-linear model (like Eq. (1)) to well-designed experimental data to decipher the limitations and possible extensions of these tests. Of note, it is now clear that these in vitro tests are robust to small measurement errors when the 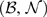 values are far from the critical bacterial curve 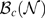. The current protocol of performing these tests at concentrations of 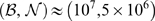 indeed satisfies this condition.

Our second objective is to shed some light on the neutrophils function in vivo. However, the relation between the in-vitro bacterial-neutrophil tests and the in-vivo strongly coupled dynamics is known to be non-trivial. This coupling may lead, for example, to recovery from an initial state at which the bacterial concentration is above its critical value by a spontaneous increase in 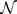. In our axiomatic formulation, such normal in-vivo dynamics clearly violates assumption A4, and indeed we cannot expect that any one-dimensional model will adequately describe this interaction. Nonetheless, there are two important implications of our construction on the in-vivo dynamics: first is the possible direct clinical implication for patients suffering from neutropenia-associated conditions as described below, and second, our construction serves as an important building block for constructing more realistic models for the common in vivo development of infections (see Models).

We propose that from a clinical perspective, the model is relevant to neutropenia-associated conditions, namely, to clinical situations in which the neutrophil bactericidal functionality is effectively constant. In each of these conditions (severe neutropenia, adhesion or bactericidal malfunction, etc.), the neutrophil supply from the bone marrow to the infected region is at maximal capacity, and so the in vivo levels and functionality of the neutrophils are essentially constant, at least for a few hours, sometimes for a few days. For example, under severe prolonged neutropenia, the neutrophil levels are fixed at very low values (below 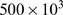 and often in the range of 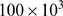 cells/mL) for several days. Moreover, a major cause of this condition are high-dose chemotherapy treatments that also damage the mucous. Such chemotherapy treatments increase the bacterial influx (Infx) and lower the 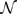 values in our model. We thus believe that our model, and in particular assumption A4, may be relevant for such specific in vivo conditions (see further discussion in Model Limitations).

Indeed, several clinical findings regarding such patients and the current recommended treatment strategies are consistent with type II behavior. First, it is clinically observed that when the white-cell blood count is low, sterile conditions and isolation, whereby patients are not exposed to any sudden influx of bacteria, do help in reducing the risk of infection [40]. This observation is inconsistent with linear and type I dynamics, where the development of infection is independent of the initial bacterial concentration. These observations are consistent with type II dynamics that predict high sensitivity to 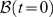, especially when 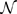 is low and Infx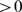, as is the case after chemotherapy. Second, it is known that patients undergoing such treatments are prone to infections and typically receive antibiotics and sometime G-CSF (growth factor that promotes neutrophil production) injections. Such treatments correspond to introducing bacterial death (by antibiotics) and an increase in neutrophil concentration (by G-CSF) in our model. We observe that with the current recommended G-CSF injection schedule, as the neutrophils recover from the nadir, their level shows an overshoot and only then settles back to normal values (see e.g., Fig. 1 in [41]). The need for such an overshoot is consistent only with type II dynamics due to the hysteresis effect: the chemotherapy acts as an external force on the neutrophils, causing these to decrease in number and then increase again to the normal values as the bone marrow recovers. This loop in the control parameter 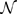 may lead to hysterises effect by which acute infections are developed even though the neutrophil count is back to normal. A sufficiently large overshoot (by G-CSF injection or by natural response) is needed to overcome such a hysterises effect.

We conclude that bistability may be a major cause of bacterial infection in neutropenic patients, and that further insights may be drawn from our model. Once the parameters of the model are calibrated, the effects of antibiotics, non-sterile conditions, and externally controlled time-dependent neutrophils can be introduced into our model and studied in more details.

### Model Limitations

The properties of the model are derived from the axiomatic biologically motivated assumptions A1–A4 (and are independent of the details of the explicit model Eq. (1), see Mathematical Remarks). While assumptions A1 and A2 seem to be common to a variety of bacterial strains, assumption A3, by which the kill rate is monotonic with 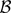, is expected to be more strain-specific; it is expected to be universally true at low concentrations, but at high concentrations of bacteria strains that form spatial structures [42] it may fail. For such strains, we expect to see more complex behavior at these high concentrations (which may still be clinically relevant). Notice that nonetheless, the division into type I and II behaviors at low concentrations is still valid.

Assumption A4 is the most restrictive, as it excludes many important in vivo effects such as time-dependent environmental variations, coupling to the neutrophil dynamics, coupling to other bacterial strains and coupling to other systemic feedback mechanisms of the body. For example, the toxic effect of high levels of neutrophils and the accompanying feedback mechansims of anti-inflamatory agents (see e.g. [7], [24]) do not enter the model. Thus, the regime of robust stable dynamics at high neutrophil concentrations of the in-vitro model (at 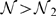) is likely to be irrelevant for the in-vivo dynamics when 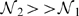. We are currently expanding our model to include some of these effects. Nonetheless, we listed several clinically relevant scenarios for which, for limited time scales, assumptions A1–A4 appear to be reasonable. In particular, we believe that both the in vitro and in vivo dynamics in the neutropenic-associated conditions fall into this category.

In the in vitro setting, these assumptions can be experimentally tested. It is possible to repeat the experiments of Li et al. [11], [12] with other bacteria and other phagocytes to examine the universality of our model. Moreover, it is also possible to measure how the suspension's content deteriorates with time and how neutrophil function changes with time. Such experiments can thus determine the time scale for which this simple model is valid and provid additional insights regarding the in vivo dynamics (see Models).

Testing these assumptions in the in vivo settings of neutropenic-associated conditions in humans is clearly more difficult–we may need to resort to Occam's razor principle by which the simplest plausible model is chosen. We did provide some circumstantial evidence for the plausibility of our model and the impossibility of the simpler linear model or type I behavior. Nonetheless, we must point out that even under these restricted conditions of constant neutrophil number and function, other dynamic variables (such as macrophages, cytokines, opsonins, anti-bactericidal factors), spatial effects associated with local infections (especially in the case of deep burns) and other temporal effects associated with the full body dynamics (like fever) may influence the bacterial growth and killing term and thus violate the A4 assumption. Here, we think of all of these as having secondary effects; nevertheless it is clear that their impact should be further studied with a specific neutropenic-associated condition in mind. Well-designed experiments in animal models may supply more insights regarding the significance of such effects in a systematic manner.

### Models

Here we discuss some of the properties of the non-linear model: we first explain in more detail how we construct the bifurcation diagrams and what kind of information can be extracted from them, then we discuss the role of the bacterial influx term (biologically and mathematically) and finally, we remark on the generality of our results and on their role as a building block in higher dimensional models.

### Bifurcation Diagrams and Hysteresis

The non-linear model (Eq. (1)) is one-dimensional and has bounded growth together with first-order elimination. Thus, the bacterial concentration always settles to some equilibrium point (EP) of Eq. (1), that is, to a finite 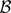 value for which the right hand side of Eq. (1), which we denote hereafter by 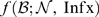, vanishes (see Fig. 1c). In particular, under natural bacterial dynamics, with no neutrophils or influx (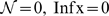), for any non-trivial initial concentration, the bacterial growth curve limits to the maximal capacity, corresponding to the infectious state: 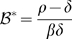 (thus, we always take 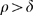). To describe the 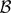 dynamics for general 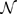, we study how the EPs depend on 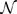. Figs. 1 and 2 present our findings as bifurcation diagrams, showing how the EP location and stability vary with 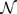. Solid curves in these figures correspond to stable EPs whereas dashed curves correspond to unstable ones. The transient dynamics is also shown in these figures. Since 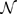 is constant, the 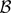 dynamics occur along vertical lines in this 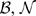 plane. The direction of motion is represented in the figures by arrows. Thus, the arrows point toward stable branches of EPs (solid curves) and away from unstable branches of EPs (dashed curves) (see Fig. 1c,d for the corresponding time-flow plot of 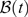 vs 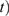. The blue-red regions are marked to stress that the EP branches separate regions for which the initial bacterial population grows (red) from regions where it decreases in size (blue).

Moreover, the bifurcation diagrams clearly show the differences between the type I and type II dynamics: the type I behavior has a single stable EP for all neutrophil levels. When Infx 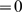, this stable branch of EPs decreases to zero as 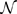 is increased beyond 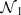. Thus 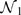 is a bifurcation point when Infx 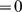; for (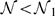), the origin is an unstable EP, at 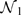 this unstable branch meets the stable EP branch that emerges from the maximal capacity value, and at (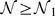), the origin becomes a stable EP. When 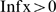, the origin is no longer an EP, and the stable EP branch simply decays smoothly towards zero as 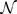 increases (mathematically we can say that the bifurcation point 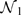 disappears in a symmetry-breaking bifurcation). Notice that for small 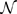, the stable branch corresponds to an infectious state, whereas for large 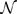 it corresponds to a healthy state (which is non-vanishing yet small when 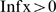). In the type I regime, the transition between a healthy state and an infectious state as the neutrophil level is changed is gradual. Moreover, in such a regime, the neutrophils always determine the eventual state of the bacteria (independent of 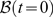).

The bacterial dynamics in a type II regime (Fig. 1b, 2b) depends more dramatically on the neutrophil level and, for a range of neutrophil levels, on the initial bacterial concentration. Here we have two bifurcation points 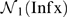 and 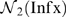, and both bifurcation points exist for 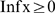 (see below for more details). For 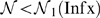, the bacteria always approach the stable EP that corresponds to high bacterial level (i.e., to an infectious state). For 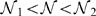 there are three EPs forming bistability: the stable high level infectious state, the stable low level healthy state, and an intermediate critical unstable state 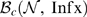. If 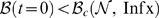, then the bacterial level decreases towards the healthy state, whereas if 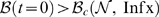, the bacterial level increases towards the infectious state. Namely, depending on the initial infection severity, 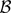 may either decrease or increase in size. When 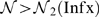, there is one stable solution that corresponds to a healthy state.

At 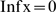, the model type is determined by the non-linear stability of the origin (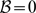) at the critical neutrophil level 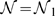: if the EP is non-linearly stable (i.e., 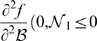), the model is of type I, and it is of type II if the EP is non-linearly unstable. For sufficiently small 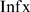, the model type remains the same as for 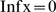. A small calculation of this non-linear stability coefficient at 
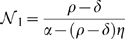
 shows that type I appears when 
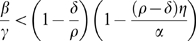
, and type II follows when the inequality is reversed (see Fig. 3). More generally, the appearance of exactly two types of models at low concentration values is not accidental: this is the simplest typical behavior for models satisfying assumptions A1–A4.

The second bifurcation point is located at 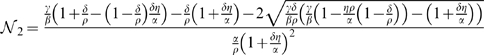, and 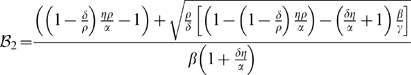. It can be shown that 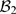 is negative in the type I dynamics and positive in the type II dynamics. If one assumes that 
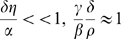
, and 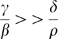 (as in Fig. 1) the above expressions simplify to: 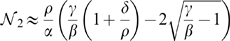, 
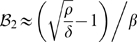
. Namely, 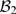 depends mainly on the natural bacteria growth curve, whereas 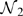 depends mainly on the ratio of linear growth vs. linear kill and on the saturation rates of the natural growth vs. the kill term.

Near the critical neutrophil value 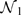, the linear model and the non-linear model at type I and type II regimes provide three distinct behaviors for low initial bacterial concentrations. Thus, a careful in vitro experiment near 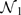 can distinguish between the three models.

Note that hysteresis is realized only in the type II dynamics: starting with a healthy state (such that 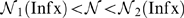 and 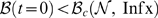 is small and under control), the result of a slow decrease in 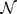 below 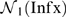 followed by a slow increase of 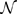 to the same initial healthy 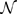 value may depend critically on the manner in which 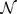 is varied. If at the end of this 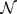 cycle 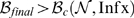, this state will evolve to a full-blown infectious state. A further increase in 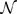 or other measures, such as antibiotics (e.g., decreasing 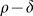), must be applied to avoid the onset of an acute infection. Note that a similar cycle of 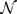 in either type I regime or in the linear model will always result in a full recovery to the healthy state with no need for intervention. Notably, the hysteresis mechanism of the type II dynamic is robust: small changes in the parameters or even addition of other small terms to the right hand side of Eq. (1) will shift the bifurcation points 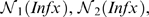 but will not destroy them.

### Bacterial Influx

While bactericidal activity experiments are performed with no bacterial influx, such influx is ubiquitous and arises naturally in the in vivo setting. For example, the digestive system barriers have to fight the bacteria from the gut that are constantly attempting to penetrate the body [1]. Mathematically, the introduction of a small influx to Eq. (1) breaks the symmetry of the model, destroying the trivial branch of equilibrium 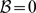 and deforming the bifurcation diagrams as shown in Fig. 2; this deformation is mild for the type I dynamic, but has very interesting implications for the type II regime, as described next. Most importantly, note that the main conclusion regarding the zero-influx model–namely, the existence of bistability for a range of neutrophil values only in the type II regime, is still valid.

Two interesting features in the type II regime emerge. First, the unstable branch 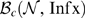 moves downward (Figs. 2b, dashed curves) as the influx is increased. Hence, the interval of initial bacterial concentration in which the bacteria are under control shrinks (for a fixed neutrophil concentration in the bistable range). This suggests that if the neutrophil level is kept constant and there is an increase in bacterial influx, the susceptibility of the patient to an acute infection increases. In fact, this happens in cemotheraphy patients, where the neutrophils are fixed at the nadir (due to the impact of the treatment on the bone marrow) and the influx is increased (due to the impact of the treatment on the barriers) [40]. Second, the influx shift of the critical neutrophil level is abrupt (Fig. 2b-insert)–the bifurcation point 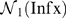 is given by 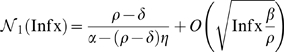, and the corresponding critical bacterial level is proportional to 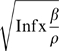 (provided that 
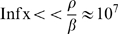
 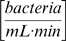 for the figure parameters, with 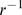 taken as the time scale and 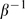 as the bacterial scale).

Biologically, this implies that near sterile conditions (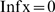), tiny changes in the bacterial influx lead to dramatic changes in the critical neutrophil value that is needed to overcome the tiniest infection; a fourfold decrease in 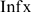 leads to only a twofold decrease in the influx-shift of the critical neutrophil levels and to a twofold decrease in the observed bacterial concentration near criticality. The effect of such sensitivity on the onset of infection is expected to be especially dramatic when the neutrophils are at low counts, near the critical value 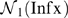.

Mathematically, this singular dependence on 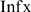 is a result of the symmetry breaking, changing the bifurcation point from transcritical to a saddle-node bifurcation. In fact, assumption A3 implies that this is the only possibility, and that this property is independent of the details of the explicit model.

### Some Mathematical Remarks

Are there other possible models to consider for the one-dimensional bacterial dynamics? It is possible to show that under assumptions A1–A4, the type I and II behaviors are the simplest possible. Other models that satisfy A1–A4 may exhibit regimes in which additional multi-stability branches at high bacterial concentrations emerge. Nevertheless, below a certain bacterial concentration, all robust models that satisfy A1–A4 must exhibit either type I or type II behavior. For example, if we replace the natural bacteria dynamic part of the model (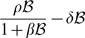) with e.g., the logistic, (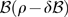), or Gompertz [36] terms, the model will result in the same qualitative dynamics.

Finally, from a mathematical perspective, at low bacterial concentrations, the division into the type I and II regimes (namely the existence of the unstable positive branch 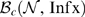 in regime II and its disappearance in regime I) appears whenever the coefficient of the quadratic term (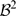) in the Taylor expansion of 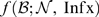 near 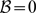 at the bifurcation point 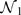 changes sign as the parameters are varied. Thus, in particular, models with a kill term that has no saturation in 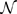 (i.e., no predator interference term, violating part of assumption A3, as in [27]), or even models for which the bacteria's natural dynamics is not limited yet has some non-linear components (violating A1), can still exhibit the type I and type II dichotomy at low concentrations. For example, if we take in model (1) 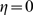 and 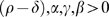, we still get the division into the two regimes. If we further set 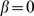, we get only the type II regime. The reasoning for stating assumptions A1–A4 is thus not mathematical–these are biologically driven assumptions that are reformulated in mathematical terms.

### Some Mathematical Remarks Regarding the In Vivo Dynamics

The characterization of the principles governing the in vitro bacterium-neutrophil dynamics may be viewed as a careful examination of a building block to be utilized in other higher dimensional models that describe various aspects of the in vivo dynamics. Indeed, all of the population type models (that neglect spatial and stochastic effects) for the in vivo dynamics include an equation which describes how the rate of change in the bacterial concentration depends on the bacterial concentration, on the phagocyte concentration and, possibly, on other factors that enter the model (see e.g. [24], [26], [43]). Thus, the model presented here may serve as a solid building block for these more complex models that are often derived in a phenomenological fashion. In particular, it is now established that bistability of the bacterial dynamics arises in nature. Thus, multidimensional models that are built upon this bistable building block and therefore exhibit rich dynamics (e.g. [27]) now have concrete reasoning for introducing this ingredient.
